# Selection of highly efficient sgRNAs for CRISPR/Cas9-based plant genome editing

**DOI:** 10.1038/srep21451

**Published:** 2016-02-19

**Authors:** Gang Liang, Huimin Zhang, Dengji Lou, Diqiu Yu

**Affiliations:** 1 Key Laboratory of Tropical Plant Resources and Sustainable Use, Xishuangbanna Tropical Botanical Garden, Kunming, Yunnan 650223, China; 2 School of Life Sciences, University of Science and Technology of China, Hefei, Anhui 230027, China; 3 University of Chinese Academy of Sciences, Beijing 100049, China

## Abstract

The CRISPR/Cas9-sgRNA system has been developed to mediate genome editing and become a powerful tool for biological research. Employing the CRISPR/Cas9-sgRNA system for genome editing and manipulation has accelerated research and expanded researchers’ ability to generate genetic models. However, the method evaluating the efficiency of sgRNAs is lacking in plants. Based on the nucleotide compositions and secondary structures of sgRNAs which have been experimentally validated in plants, we instituted criteria to design efficient sgRNAs. To facilitate the assembly of multiple sgRNA cassettes, we also developed a new strategy to rapidly construct CRISPR/Cas9-sgRNA system for multiplex editing in plants. In theory, up to ten single guide RNA (sgRNA) cassettes can be simultaneously assembled into the final binary vectors. As a proof of concept, 21 sgRNAs complying with the criteria were designed and the corresponding Cas9/sgRNAs expression vectors were constructed. Sequencing analysis of transgenic rice plants suggested that 82% of the desired target sites were edited with deletion, insertion, substitution, and inversion, displaying high editing efficiency. This work provides a convenient approach to select efficient sgRNAs for target editing.

With the rapid development of genome sequencing technology and bioinformatics, increasing genome-scale genetic elements are annotated in different species. A major goal for biologists is to characterize the biological functions of those genetic elements. Genome-scale loss-of-function mutants with T-DNA insertions have provided a wealth of information in diverse plant model systems, such as *Arabidopsis* and rice. However, due to the low coverage, a number of genes are still not targeted by T-DNA. RNA interference (RNAi) is a predominant method for suppressing a certain specific gene, but its utility is limited by the incompleteness of protein depletion by RNAi and potential off-target effects.

Recently, sequence-specific nucleases have been developed as an effective tool to perform genome editing, such as zinc-finger nucleases (ZFNs) and transcription activator-like effector nucleases (TALENs). However, due to both ZFN and TALEN technologies involving engineering of DNA binding domain for individual targeting applications, significant effort and expertise in molecular cloning are required. More recently, the CRISPR (clustered regularly interspaced short palindromic repeat)-associated (Cas) endonuclease Cas9 has been confirmed as a powerful tool for genome editing in diverse plant species^1^,^2^,^3^,^4^,^5^,^6^,^7^,^8^,^9^,^10^,^11^. The CRISPR-Cas9 system contains a *Cas9* gene and its corresponding CRISPR array. A characteristic CRISPR array consists of repetitive sequences (repeats) interspaced by short stretches of nonrepetitive sequences (spacers) derived from short segments of foreign genetic material (protospacers). The CRISPR array is transcribed and processed into short CRISPR RNAs (crRNAs). The crRNA and the trans-activating crRNA (tracrRNA) form a crRNA:tracrRNA duplex which directs the cleavage of cognate DNA sequences upstream of the appropriate protospacer-adjacent motifs (PAM)^12^,^13^. A single guide RNA (sgRNA) consisting of a synthetic fusion of crRNA and tracrRNA can be incorporated into the Cas9/sgRNA complex to cleave its target sequence preceding a 5′-NGG-3′ PAM sequence. In contrast to ZFN and TALEN, the CRISPR/Cas9-sgRNA system recognizes its target sites through Watson-Crick base pairing. The spacer sequence of sgRNA is complementary to and cleaves the target DNA by guiding the Cas9 protein. A single guide RNA can be functionally expressed under small nuclear RNA (snoRNA) promoters such as U6 or U3^14^,^15^.

A major concern of CRISPR/Cas9-sgRNA system is the editing efficiency and specificity of sgRNA. Recently, two groups developed web tools for design of highly specific sgRNA in plants^16^,^17^, which provides a number of highly specific guide sequences for a given gene. However, it is still unknown whether a certain candidate guide sequence is efficient or inefficient in plants.

The construction of multiple mutants is often required for functional research of multiple duplicated genes, which is extremely time-consuming, particularly for the closely-linked genes. Given that multiple sgRNAs can be expressed simultaneously, editing multiple genomic loci is allowed. Recent reports showed that up to 8 target sites can be edited simultaneously in rice^4^,^9^.

In the present study, criteria for efficient sgRNAs were instituted according to the assessment of nucleotide composition and secondary structure of sgRNAs. At the same time, we invented a new strategy to construct multiple target editing CRISPR/Cas9-sgRNA system. Experimental test of 21 sgRNAs in rice plants demonstrated a high editing efficiency in the expected target sites.

## Results

### Design of efficient sgRNAs

The CRISPR/Cas9-sgRNA system has been adapted for facilitating genome editing in eukaryotic cells. Although this system can be programmed to virtually cleave any sequence preceding a 5′-NGG-3′ PAM sequence, it does not always succeed with regard to all sites predicted to be targeted^9^,^18^. The major concerns of CRISPR/Cas9-sgRNA system are the target specificity and efficiency. For a given gene, tens or hundreds of NGG containing sites can be candidate editing loci. Although several online tools were developed for highly specific sgRNA selection^16^,^17^, the tools for the evaluation of sgRNA editing efficiency in plants are still lacking. Unlike the screening libraries in animal cell lines, genome editing in plants takes long time to obtain transgenic products. Thus, criteria that can be used to distinguish efficient and inefficient sgRNAs are of great utility for avoiding generation of non-edited transgenic plants resulting from inefficient sgRNAs.

**Figure 1:**
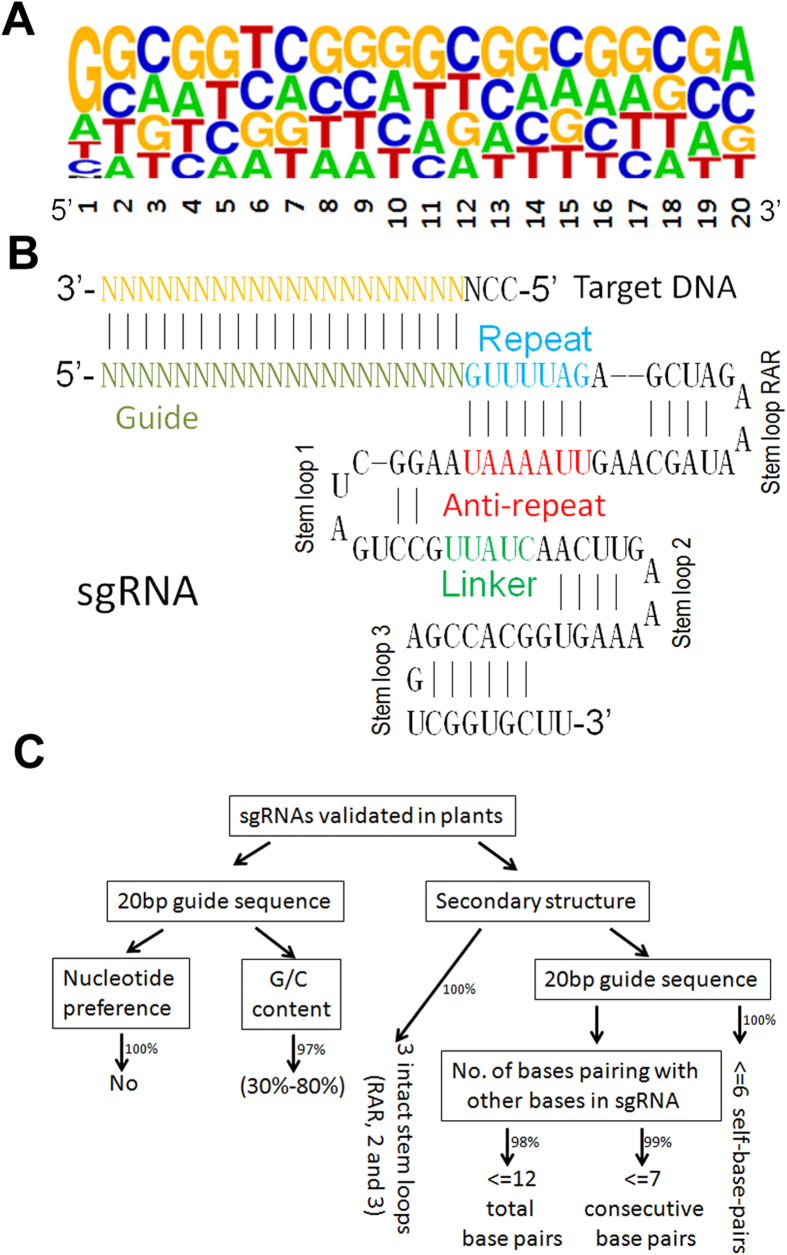
Design criteria for efficient sgRNA in plants. (**A**) Logos showing the sequence preference of sgRNAs which were experimentally validated previously. The height of the nucleotides represents the nucleotide frequency. (**B**) Schematic representation of the sgRNA secondary structure. Watson-Crick and non-Watson-Crick base pairs are indicated by black lines, respectively. Disordered nucleotides are boxed by dashed lines. (**C**) Pipeline for institution of design criteria for efficient sgRNAs.

To institute criteria to design efficient sgRNAs, we collected and analyzed those sgRNAs which have been validated in plants (Supplemental Table 1). Several groups analyzed the nucleotide composition of sgRNAs used in animal and identified a few nucleotide preferences^18^,^19^,^20^. However, the nucleotide preferences were not found in plant sgRNAs. We saw no statistically-significant difference for the nucleotide composition in each of all 20 positions, namely, no nucleotide preference was observed (Fig. 1A), implying a difference between animal and plant sgRNAs. G/C content has been thought as a key factor affecting sgRNA editing efficiency^9^,^18^,^19^,^21^. Our analysis revealed that 97% of sgRNAs have a G/C content between 30% and 80%.

It is well known that one sgRNA functions by the interaction of its secondary structure with the Cas9 protein *in vivo*^22^. Ma *et al.*^9^ suggested that the secondary structure of sgRNA may interfere with the editing efficiency. Therefore, to establish link between secondary structure and editing efficiency of sgRNAs is necessary. The sgRNA contains crRNA- and tracrRNA-derived sequences connected by an artificial tetraloop. The crRNA sequence consists of guide (20nt) (also referred to spacer) and repeat (12nt) region, whereas the tracrRNA sequence consists of anti-repeat (14nt) and three tracrRNA stem loops (Fig. 1B). The repeat and anti-repeat region (stem loop RAR) triggers precursor CRISPR RNA (pre-crRNA) processing by the enzyme RNase III and subsequently activates crRNA-guided DNA cleavage by Cas9^14^. The analysis of crystal structure of Cas9-sgRNA-DNA revealed that stem loop 1 is crucial for the formation of the functional Cas9-sgRNA-DNA complex whereas stem loop 2 and 3 promote the stable complex formation and hence improve the *in vivo* activity^22^. Since the guide sequence is variable in an sgRNA, the final secondary structure should vary with the guide sequence. Assessment of sgRNA secondary structures found that all sgRNAs validated in plants have intact stem loop RAR, as well as intact stem loop 2 and 3, implying that these 3 stem loop structures are crucial for genome editing. In contrast, 82% of sgRNAs lose their stem loop1, implying that stem loop 1 is not related with editing efficiency. It is noteworthy that 83% of guide sequences contain at least one base pairing with the other nucleotide(s) of sgRNAs (Supplemental Table 1). Because a stable complex between the guide sequence and the other bases can affect the base pairing of guide sequence with its target DNA, we further analyzed the number of pairing-bases, finding that 98% of guide sequences have no more than 12 bases paring with the other bases of sgRNA and 99% of guide sequences have no more than 7 consecutive base pairs (CBPs). In addition, internal base pairs (IBP) in the guide sequence also may interfere with its target recognition. Statistical analysis indicates that 35% of guide sequences contain at least one IBP and the highest IBP number is no more than 6.

Based on these analyses above, criteria for selection of efficient sgRNAs were instituted (Fig. 1C). (1) G/C content between 30% and 80%; (2) intact secondary structures except for stem loop 1; (3) no more than 12 total base pairs (TBPs) and no more than 7 CBPs between guide sequence and the other sequence; (4) no more than 6 IBPs.

### An alternative assembly system for CRISPR/Cas9-sgRNA multiple-targeting

The type II CRISPR/Cas9-sgRNA system comprises two key components, Cas9 and sgRNA. Several types of codon-optimized Cas9 genes have been validated in plant genome editing^1^,^2^,^3^,^7^,^9^,^23^. Here, we selected a plant-codon optimized *Cas9* gene (*Cas9p*) (Ma *et al.*^24^ 2015). To perform the expression of multiple sgRNA cassettes in the monocot and dicot plants, U3 and U6 small RNA promoters from rice (OsU3, OsU6a, OsU6b, and OsU6c)^3^,^7^,^9^,^23^ and *Arabidopsis* (AtU3b, AtU3d, AtU6-1, and AtU6-29)^25^ can be chosen.

T-DNA is commonly used for delivery of DNA. We developed a restriction enzyme based system to assemble *Cas9* and multiple sgRNAs into a T-DNA. This system consists of two modules for the assembly of multiple sgRNA cassettes and the *Cas9* gene. The first module is an intermediate vector (pSAK2) (Supplemental Fig. 1) which contains a multiple clone site consisting of eleven regular restriction enzyme sites and hence can load up to ten sgRNA cassettes in theory. The pSAK2 vector is derived from the pBluescript II KSII (+) and retains the *Lac* Z marker gene which can be used to help selection for positive clones carrying the sgRNA expression cassettes. The second module contains a set of four binary vectors (Supplemental Fig. 2) adapted from those of Ma *et al.*^9^, each one of which possesses one *Cas9p* gene driven by the cauliflower mosaic virus 35S promoter (P_35S_) or the *Zea may* ubiquitin promoter (P_ubi_) and a multiple clone site containing two restriction enzyme sites (*Spe* I and *Asc* I) used for accepting the sgRNA cassettes from the first module.

**Figure 2:**
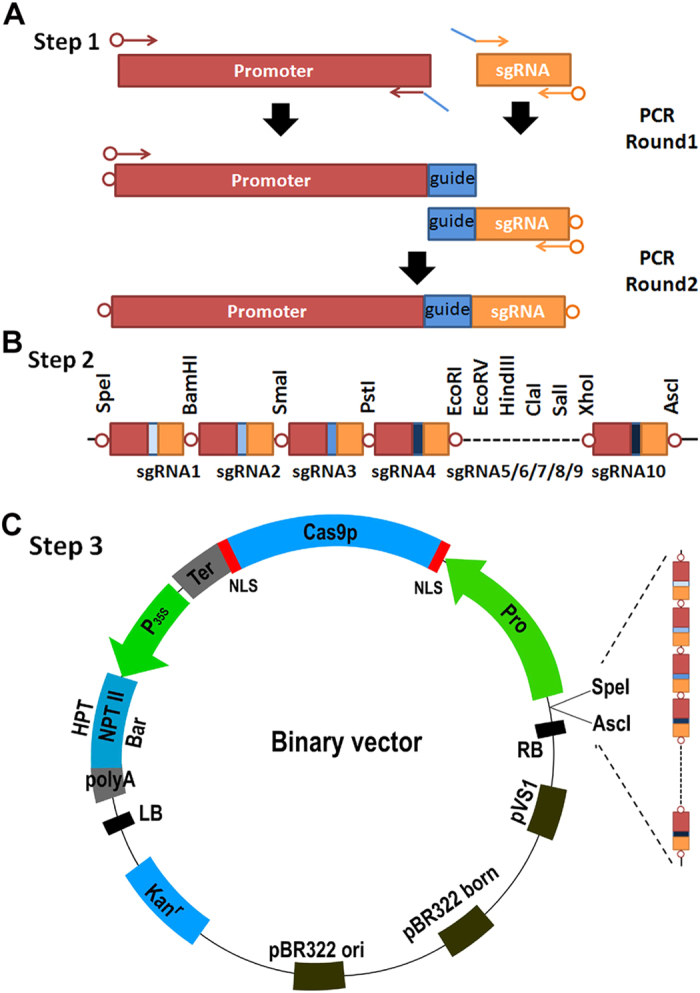
Construction strategy of CRISPR/Cas9-sgRNA system. (**A**) Generation of an intact sgRNA cassette. Guide sequence (blue bar) containing chimeric primers were used to perform over-lapping PCR to generate an sgRNA cassette. The arrow-circle indicates the public primer with specific restriction enzyme sites. (**B**) Multiple clone sites of the intermediate vector pSAK2. 11 regular restriction enzyme sites are included, which can be used for assembly of up to 10 sgRNA cassettes in theory. (**C**) Structures of the binary vectors based on the pCAMBIA1300 backbone. HPT, Bar, and NPT II encode hygromycin B phosphotransferase, PPT acetyltransferase and neomycin phosphotransferase II, respectively. Two restriction enzyme sites, *Spe* I/*Asc* I, were provided for the entry of multiple sgRNA cassettes.

The first step is to construct sgRNA expression cassettes containing the guide sequences. The guide sequences can be easily integrated into sgRNA expression cassettes by overlapping PCR with guide sequence-containing chimeric primers (Fig. 2A). At the same time, ten pairs of public primers, which can pair with the 5′-terminal of promoters and the 3′-terminal of sgRNA and contain specific restriction enzyme sites, are available (Supplemental Table 2). The public primers of interest should be selected according to the desired order of sgRNA expression cassettes. After two rounds of PCR, an intact sgRNA expression cassette with two different restriction enzyme sites in the 5′ and 3′ ends can be produced (Fig. 2A). The second step is to clone different sgRNA expression cassettes into the intermediate vector pSAK2 (Fig. 2B). The third step is to digest pSAK2-sgRNA(s) by *Spe* I/*Asc* I and then ligate the sgRNA(s) into the final binary vectors (Fig. 2C; Supplemental Fig. 2).

### Highly efficient genome editing

Having instituted the criteria for selection of efficient sgRNAs in plants, subsequently we performed experimental test in rice plants. To reduce potential off-targets, the guide sequences with a high specificity to desired target sites were selected from the CRISPR-PLANT Database^16^. Then, 21 guide sequences (Guide1 to Guide 21) which agree with the criteria were used for the next test (Supplemental Table 3). To further confirm whether the criteria are reliable, we also designed two guide sequences (Guide22 and Guide23) against the criteria as potential inefficient sgRNAs. sgRNA22 contains up to 7 IBPs in the predicted secondary structure and sgRNA23′s G/C content is less than 30% (Supplemental Table 3; Supplemental Fig. 3). Guide 1 to 23 are predicted to target 11 encoding genes (Supplemental Table 3) and assembled into sgRNA1 to sgRNA23, respectively. The sgRNA cassettes were further cloned into the binary vector, pMH-SA (Supplemental Fig. 2), by the aforementioned method. At last, 12 expression constructs with one or multiple sgRNA cassettes were generated (Table 1) and then used for rice transformation.

**Figure 3:**
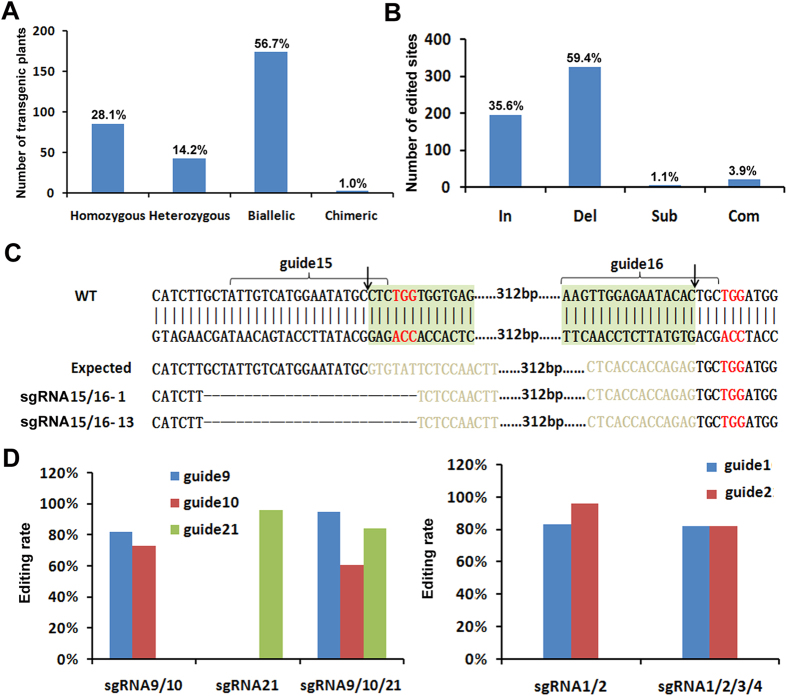
Characterization of genome editing. (**A**) Editing frequency of transgenic plants. The number on the bar indicates the percentage of each genotype in all edited plants. (**B**) Editing types. The number on the bar indicates the percentage of each editing type in all edited sites. In (insertion), Del (deletion), Sub (substitution) and Com (complex). (**C**) Inversion of the DNA fragment. Shown are the amplified upstream and downstream junctions as well as their sequences. Deleted bases are indicated by dashes. Arrow indicates the predicted cleavage site. The red 3-base sequence indicates the PAM motif. Guide 15/16 sequences are indicated. (**D**) Comparison of editing rate between constructs with different number of sgRNA cassettes.

To detect the Cas9-sgRNA-mediated precise genome editing, T_0_ transgenic plants were used for sequencing analysis of desired target sites. PCR products covering predicted target sites were directly used for sequencing analysis. As expected, sgRNA 1 to 21 caused genome editing in the predicted target sites. In 371 sequenced plants (except for sgRNA22 and sgRNA23), 305 plants (82.2%) had mutations, which can be classified into homozygous (28.1%), heterozygous (14.2%), biallelic (56.7%) and chimeric (1.0%) mutations (Fig. 3A). In contrast to the 21 sgRNAs (sgRNA1 to sgRNA21) with the high editing rate, no target mutation was detected in all T_0_ transgenic plants containing sgRNA22 and sgRNA23 (20% G/C content) which do not comply with the criteria. sgRNA24^9^ is a confirmed inefficient sgRNA which contains 14 CBPs and does not agree with the criteria (Supplemental Table 3). These evidence suggested that the criteria are reliable for selection of efficient sgRNAs.

We further analyzed all 545 edited sites, finding that the editing types included insertion (35.6%), deletion (59.4%), substitution (1.1%) and complex (including at least two of insertion/deletion/substitution) (3.9%) (Fig. 3B). For the 21 efficient sgRNAs with average 74.3% editing rate, sgRNA16 has the highest editing rate (100%) and sgRNA18 has the lowest (23.3%) (Table 1). Although DNA fragment inversion event by two sgRNAs was found in animals^26^, there is no report on inversion event mediated by CRISPR/Cas9-sgRNA system in plants. We found that two out of 23 sgRNA15/16 transgenic plants contains DNA fragment inversion between their two target sites (Fig. 3C). In all of 9 two-sgRNA expressing constructs, only one caused fragment inversion, suggesting a low frequency event.

To investigate whether the number of simultaneously expressed sgRNAs affects the editing efficiency, the editing frequency was analyzed between transgenic plants with two sgRNAs (sgRNA1/2) and those with four sgRNAs (sgRNA1/2/3/4), as well as sgRNA9/10, sgRNA21, and sgRNA9/10/21. As shown in Fig. 3D, no significant difference was observed. The results suggested that the number of sgRNAs has no significant effect on the editing efficiency.

## Discussion

The CRISPR/Cas9-sgRNA mediated precise genome editing is being universally applied in diverse plant species. In the present study, we instituted criteria for efficient sgRNAs based on nucleotide composition of guide sequences and secondary structure of sgRNAs and introduced a new strategy to construct CRISPR/Cas9-sgRNA cassettes. Using rice as an example, we demonstrated that this clone strategy for assembly of multiple sgRNAs is rapid and functional, and the criteria can be used to select efficient sgRNAs from the highly specific sgRNAs.

Target specificity is a major concern for the CRISPR/Cas9-sgRNA system. High-frequency off-target mutagenesis induced by CRISPR/Cas9-sgRNA was found in human cells^27^. Several studies have investigated off-target effects caused by CRISPR/Cas9-sgRNA system in animal based on the alignment of guide sequences to the genome^28^, dCas9 ChIP-seq^29^ and GUIDE-seq^30^. The off-target effects were also detected in plant cells^3^. Genome-wide specificity analysis of candidate target sites can help avoid or reduce off-target effects^16^,^17^. Another major concern for CRISPR/Cas9-sgRNA system is editing efficiency of sgRNAs. It has been established that the PAM containing NGG consensus is necessary for Cas9-DNA binding and cleavage in a CRISPR/Cas9-sgRNA system^31^. Recent studies showed that the sgRNA editing efficiency depends on nucleotide composition of guide sequences in animals^19^,^20^. By analyzing sgRNAs which have been experimentally validated in plants, we found that most of their guide sequences have G/C content between 30% and 80% whereas no nucleotide preference was found. A recent study revealed that the guide sequence with the higher G/C content has the higher editing efficiency^9^. Based on the secondary structures of sgRNAs, criteria for efficient sgRNAs were instituted. Experimental tests revealed that all 21 sgRNAs agreeing with the criteria were efficient for target site editing. Many factors can affect the editing efficiency of sgRNAs. In addition to nucleotide composition and secondary structure, the expression levels of sgRNAs may be also involved^9^. Thus, in order to ensure efficient editing of a given gene, more than one sgRNA is recommended to be selected to target multiple sites in this gene.

Very recently, Golden Gate cloning method was used to assemble multiple sgRNA cassettes^9^,^32^,^33^, which employs the type II restriction enzyme, *Bsa* I, to design and generate distinct, non-palindromic sticky ends. This method is efficient for linking multiple sgRNA cassettes in a designed order in single reaction because the sticky ends caused by *Bsa* I can avoid self-ligation and non-compatible end ligation. The efficiency for assembly of two or three sgRNA cassettes was high whereas 6 or more sgRNA cassettes was far less efficient and often failed^33^. The *Cas9* gene and *Zea may* ubiquitin promoter sequences contain most of regular restriction enzyme sites, which limits the utility of regular restriction enzymes in the assembly of multiple sgRNA cassettes. Here, we developed two modules to construct CRISPR/Cas9-sgRNA system containing multiple sgRNA cassettes. Both modules employ common restriction enzymes to perform molecular cloning. In theory, our method can assemble up to 10 sgRNA cassettes by the use of 11 different restriction enzyme sites provided in the pSAK2. Our two modules comprise an efficient, inexpensive, time-saving, user-friendly, multifaceted, extensible toolkit for the construction of CRISPR/Cas9/sgRNA system targeting multiple genome sites.

The efficient editing of target genes in transgenic plants can provide researcher with desired mutants, which will accelerate the progress of gene function dissection. We confirmed that the sgRNAs complying with the criteria were efficient for target gene editing. Both this work and Ma *et al.*’s^9^ confirmed that at least two same snoRNA promoters can be used simultaneously to drive different sgRNAs. Our work also revealed that the number of sgRNA cassettes has no effect on the editing efficiency of sgRNAs. It is noteworthy that up to 84.8% of edited plants contained loss-of-function gene mutations (i.e., biallelic or homozygous mutations) in the T_0_ transgenic plants and they can be used directly for functional analysis. In our two-sgRNA expression plants, both targets sites can be edited simultaneously, which also caused deletion or inversion of DNA fragment between two target sites. The editing of both target sites will facilitate the gene correction via homologous recombination by providing a mutant plant with wild type DNA fragment donor. Altogether, our toolbox for sgRNA design criteria and assembly of multiplex CRISPR/Cas9-sgRNA system provides researchers with a new approach to efficiently edit one or multiple target sites and perform genetic improvement.

## Methods

### Design of the CRISPR/Cas9-sgRNA-related vectors

The pSAK2 (Supplemental Fig. 1) was derived from the pBluescript II KSII and introduced into an *Asc* I restriction enzyme recognition site in the multiple clone site. 11 restriction enzyme sites between *Spe* I and *Asc* I can be used for assembly of multiple sgRNAs. Four binary vectors, pYLCRISPR/Cas9P_35S_-H, pYLCRISPR/Cas9P_35S_-N, pYLCRISPR/Cas9P_ubi_-H, and pYLCRISPR/Cas9P_ubi_-B^9^, were modified by the remove of *ccdB* gene and introduction of *Spe* I and *Asc* I to generate pDH-AS, pDK-SA, pMH-SA, and pMB-AS (Supplemental Fig. 2), respectively.

### Assembly of multiple sgRNAs

Vectors (pYLsgRNA-OsU3, pYLsgRNA-OsU6a, pYLsgRNA-OsU6b, and pYLsgRNA-OsU6c for rice and pYLsgRNA-AtU3b, pYLsgRNA-AtU3d, pYLsgRNA-AtU6-1, and pYLsgRNA-AtU6-29 for Arabidopsis)^9^ with four rice snoRNA promoters and four Arabidopsis snoRNA promoters were used for templates in the overlapping PCR. After two rounds of PCR, as described in Fig. 2A, by specific primers and public primers, specific sgRNA cassette products with specific restriction sites in the both ends were produced, which then were cloned into the pSAK2 vector. One or multiple sgRNA cassettes between the *Asc* I and *Spe* I of pSAK2 were cloned into the binary vector by digestion and ligation. When the OsU3 promoter containing an *Spe* I cutting site is chosen, the *Xba* I (an isocaudarner of *Spe* I) cutting site can be chosen to avoid the digestion of OsU3 promoter.

### Analysis of nucleotide composition and secondary structure

All sgRNAs which were validated experimentally in plants^1^,^2^,^3^,^4^,^5^,^6^,^7^,^8^,^9^,^10^,^11^ were used for analysis. The logo for nucleotide frequency in each position was produced in weblogo (http://weblogo.berkeley.edu/logo.cgi). G/C content for each guide sequence was calculated by a Perl script. The secondary structure for each sgRNA was predicted in mfold 2.3^34^.

### Plant Transformation

*Oryza sativa spp. japonica* was used for transformation. The CRISPR/Cas9 constructs were introduced into *Agrobacterium tumefaciens* strain EHA105 by electroporation. Transformation of rice was described previously^35^.

### Mutation Detection

Genomic DNA was extracted from leaves of T_0_ transgenic rice plants and used for templates for PCR by primers flanking the predicted target sites. The PCR products were sequenced directly by specific primers. For low quality of sequencing results, PCR products were cloned into a plasmid vector and 4–6 single clones were further sequenced. Samples with heterozygous and biallelic mutations were decoded using the Degenerate Sequence Decoding (DSD) method^24^.

## Additional Information

**How to cite this article**: Liang, G. *et al.* Selection of highly efficient sgRNAs for CRISPR/Cas9-based plant genome editing. *Sci. Rep.* **6**, 21451; doi: 10.1038/srep21451 (2016).

## Supplementary Material

Supplementary Information

## Figures and Tables

**Table 1:** Genotypes and editing types resulted from each construct.

Constructs	Guide	Promoter	Genotypes					Eidting types					Editing		
			WT	Hom	Het	Bia	Chi	WT	In	Del	Sub	Com	Total	Edited	Editing rate
sgRNA1/2	guide1	OsU6a	1	4	2	5	0	4	5	13	0	2	24	20	83.3%
	guide2	OsU6b	0	5	1	6	0	1	1	20	0	2	24	23	95.8%
sgRNA5/6	guide5	OsU3	1	1	1	9	0	3	5	15	0	1	24	21	87.5%
	guide6	OsU6a	1	6	1	4	0	2	1	21	0	0	24	22	91.7%
sgRNA7/8	guide7	OsU3	4	0	7	6	0	15	1	17	0	1	34	19	55.9%
	guide8	OsU6a	7	4	6	0	0	20	0	14	0	0	34	14	41.2%
sgRNA9/10	guide9	OsU6a	2	3	0	3	3	4	1	17	0	0	22	18	81.8%
	guide10	OsU6b	3	1	0	7	0	6	8	8	0	0	22	16	72.7%
sgRNA11/12	guide11	OsU3	2	1	1	12	0	5	9	18	0	0	32	27	84.4%
	guide12	OsU6a	6	0	8	2	0	20	9	2	1	0	32	12	37.5%
sgRNA13/14	guide13	OsU3	2	9	0	9	0	4	25	9	0	0	38	34	89.5%
	guide14	OsU6a	9	4	2	4	0	20	8	9	0	1	38	18	47.4%
sgRNA15/16	guide15	OsU3	4	3	0	6	0	8	5	8	0	5	26	18	69.2%
	guide16	OsU6a	0	4	0	9	0	0	3	16	0	7	26	26	100.0%
sgRNA17/18	guide17	OsU3	2	4	1	8	0	5	16	5	4	0	30	25	83.3%
	guide18	OsU6a	3	0	1	11	0	23	0	6	1	0	30	7	23.3%
sgRNA19/20	guide19	OsU3	1	3	2	6	0	4	11	9	0	0	24	20	83.3%
	guide20	OsU6a	1	6	0	5	0	2	21	1	0	0	24	22	91.7%
sgRNA21	guide21	OsU6b	0	4	1	7	0	1	3	20	0	0	24	23	95.8%
sgRNA1/2/3/4	guide1	OsU6a	2	2	1	6	0	4	5	13	0	0	22	18	81.8%
	guide2	OsU6b	1	1	2	7	0	4	1	17	0	0	22	18	81.8%
	guide3	OsU6a	1	6	1	3	0	3	18	1	0	0	22	19	86.4%
	guide4	OsU6b	4	3	0	4	0	8	11	3	0	0	22	14	63.6%
sgRNA9/10/21	guide9	OsU6a	1	3	0	15	0	2	5	29	0	2	38	36	94.7%
	guide10	OsU6b	5	3	5	6	0	15	9	14	0	0	38	23	60.5%
	guide21	OsU6b	3	5	0	11	0	6	13	19	0	0	38	32	84.2%
sgRNA22	guide22	OsU6a	15	0	0	0	0	30	0	0	0	0	30	0	0%
sgRNA23	guide23	OsU6a	13	0	0	0	0	26	0	0	0	0	26	0	0%

WT (wild type), hom (homozygous), Het (heterozygous), Bia (biallelic), Chi (chimeric), In (insertion), Del (deletion), Sub (substitution) and Com (complex). Total (sequenced sites) and Edited (edited sites).
