# Supplementary material for: Selection of highly efficient sgRNAs for CRISPR/Cas9-based plant genome editing
Source: Sci Rep. 2016 Feb 19;6:21451. doi: 10.1038/srep21451 (PMC4759811; doi:10.1038/srep21451)
Supplement: Supplementary Information [file srep21451-s1.doc]

**Selection of highly efficient sgRNAs for CRISPR/Cas9-based plant genome editing**

Gang Liang^1#^, Huimin Zhang^1,2#^, Dengji Lou^1,3^, Diqiu Yu^1^*

^1^Key Laboratory of Tropical Plant Resources and Sustainable Use, Xishuangbanna Tropical Botanical Garden, Kunming, Yunnan 650223, China

^2^School of Life Sciences, University of Science and Technology of China, Hefei, Anhui 230027, China

^3^University of Chinese Academy of Sciences, Beijing 100049, China

# These authors contributed equally to this paper.

**Supplemental** **Figure 1**. Map of the immediate vector pSAK2.


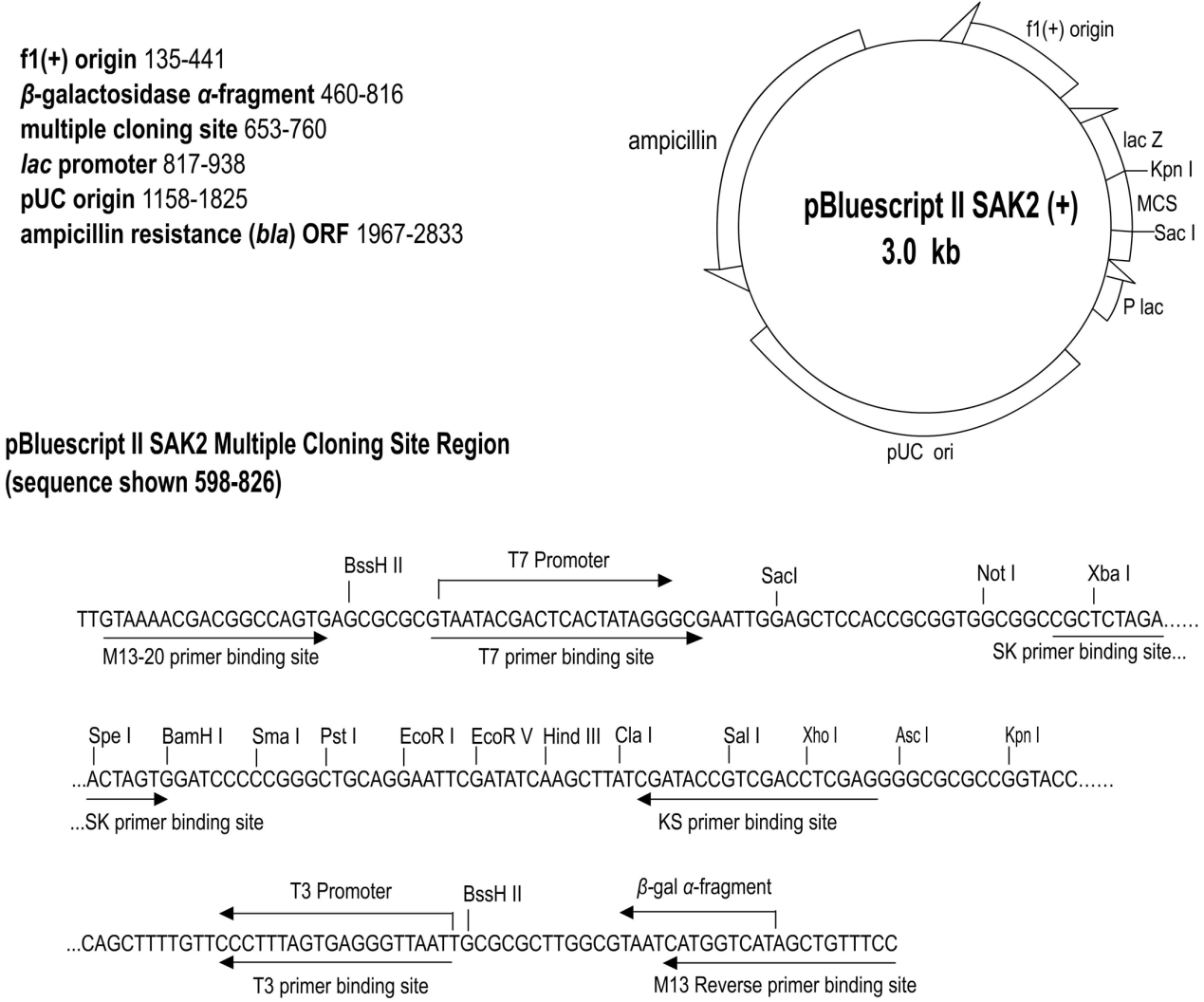


**Supplemental** **Figure 2**. Map of the four binary vectors.


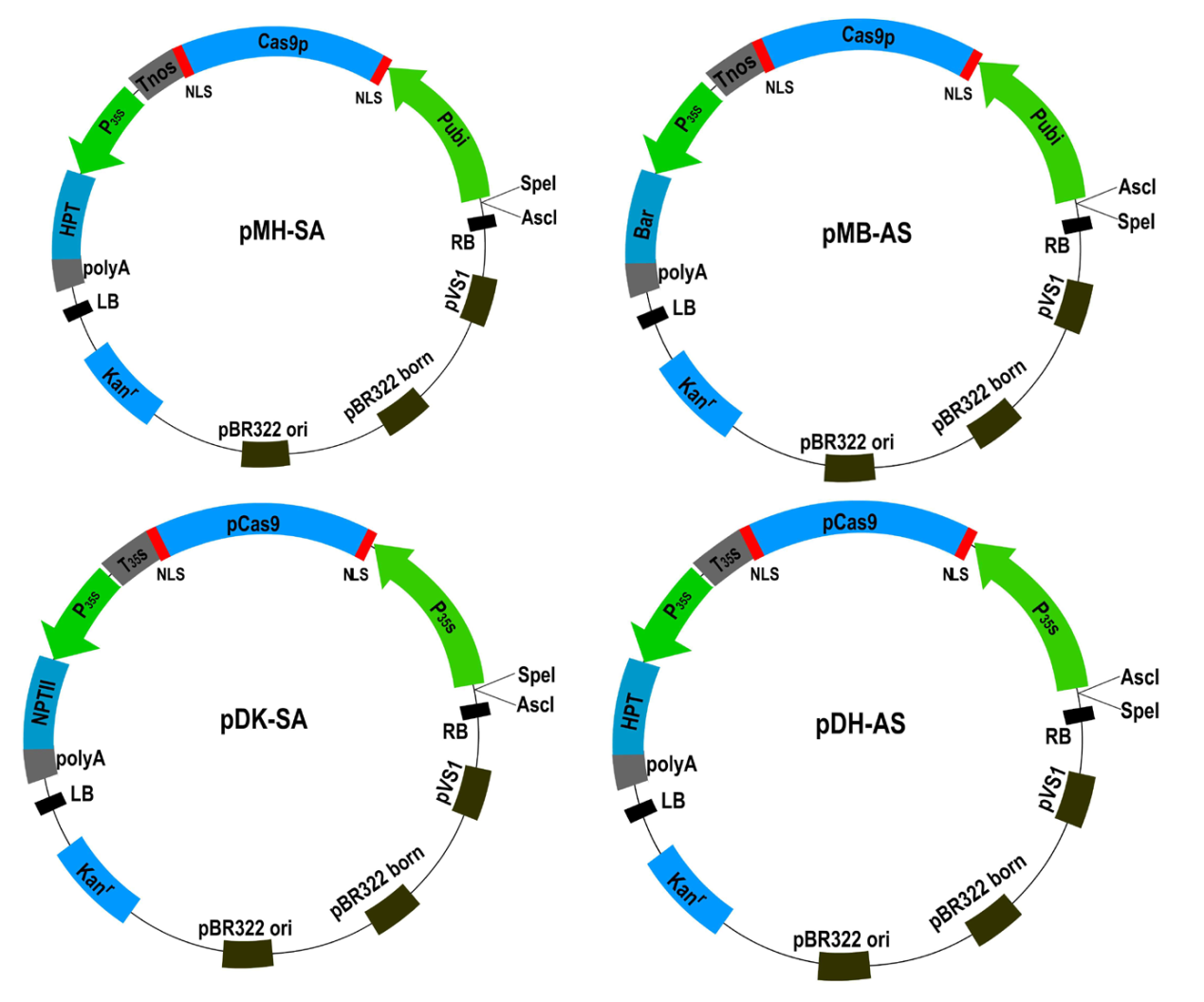


**Supplemental** **Figure** **3**. Secondary structure of sgRNA22.


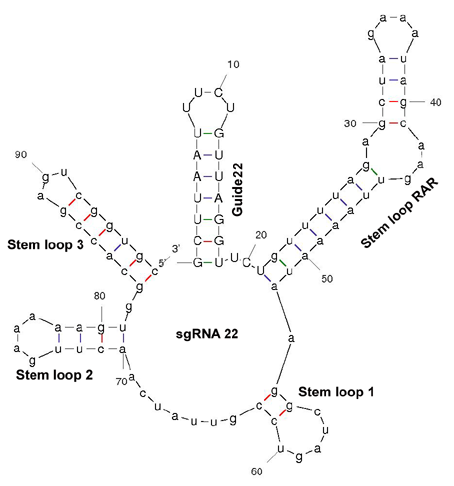


**Supplemental Table 1**. Secondary structure characteristics of plant sgRNAs validated previously.

| Guide sequence | Target sites | G/C(%) | TBP | CBP | IBP | DSL |
| --- | --- | --- | --- | --- | --- | --- |
| CGTATCTTCGGCCATGAAGC | AtADH1-A | 55% | 0 | 0 | 5 | * |
| CTTAGCCTGGCGAAAGAGTC | AtADH1-B | 55% | 0 | 7 | 4 | SL1 |
| AGAGTTTTCCCAGTTCCCGT | AtADH1-C | 50% | 0 | 2 | 0 | SL1 |
| TTGGGTCATAACGATATCTC | AtBRI1 | 40% | 0 | 6 | 0 | SL1 |
| NCCCCCATTTGCTTCAGGCC | AtCHLI1 | 60% | 0 | 4 | 0 | SL1 |
| GACATTCATAACAGAGACAT | AtCHLI2 | 35% | 0 | 5 | 0 | SL1 |
| GTTGCTCACGTAAGCGATTT | AtFLS2 | 45% | 0 | 0 | 6 | * |
| AGAGTGATTGATCTATTAAA | AtFT | 25% | 0 | 0 | 2 | * |
| GATGAGCTTCTAGCTGTTCT | AtGAI1 | 45% | 0 | 6 | 0 | SL1 |
| GCAATAGGAAGTTCTGACAA | AtJAZ1 | 40% | 0 | 3 | 0 | SL1 |
| NACAGGGTAATAGAGATAAA | AtMRS2-2 | 30% | 0 | 5 | 0 | SL1 |
| GGACTTTTGCCAGCCATGGT | AtPDS3-A | 55% | 0 | 5 | 0 | SL1 |
| GGAACAACGAGATGCTGACA | AtPDS3-B | 50% | 0 | 4 | 0 | SL1 |
| GTGATCGTACGATTAAGCTT | AtRACK1b | 40% | 0 | 3 | 5 | SL1 |
| ACCTCCGGCGTCGACATGCC | AtTT4 | 70% | 0 | 4 | 0 | SL1 |
| NGCAGATCTTGCAGAATCCT | BADH2 | 45% | 0 | 1 | 3 | * |
| AGATCGGGGAGGGGACGTAC | CDKA2 | 65% | 0 | 4 | 0 | SL1 |
| AGGTCGGGGAGGGGACGTAC | CDKB2 | 70% | 0 | 4 | 0 | SL1 |
| TTGTGCACAAGCAATTGTAC | CsPDS | 40% | 0 | 0 | 4 | * |
| GGAATTAGCGGCGGCGGCGA | DERF1 | 70% | 0 | 6 | 0 | SL1 |
| GGATGTTCACTACATGCTTG | EPSPS | 45% | 0 | 4 | 0 | SL1 |
| GCTACTTGAAGCTAGGATAA | GmDDM1 | 40% | 0 | 7 | 3 | SL1 |
| GTGTGAATGTTTATTGTGGT | GmGT4 | 35% | 0 | 0 | 2 | * |
| GGCATGGTGCGGTCTATGAG | GmMET1 | 60% | 0 | 5 | 0 | SL1 |
| GAAATCACGGTTGAGTGTGA | GmMIR | 45% | 0 | 0 | 5 | * |
| NTGGCCCACGGGGTATAAAA | MPK1-A | 50% | 0 | 0 | 4 | * |
| ATCCAGGCGACGCTGAGCCA | MPK1-B | 65% | 0 | 4 | 0 | SL1 |
| GGCGGCGGCCATGGCCATCA | MPK2-A | 75% | 0 | 5 | 0 | SL1 |
| GAACCCGGTCGCCTCAAGGA | MPK2-C | 65% | 0 | 4 | 0 | SL1 |
| GAATGCGCAGACTCGTCAGG | MPK2-D | 60% | 0 | 4 | 0 | SL1 |
| AGATGTCGTAGAGCAGGTAC | MPK5-A | 50% | 0 | 4 | 0 | SL1 |
| CTACATCGCCACGGAGCTCA | MPK5-B | 60% | 0 | 5 | 0 | SL1 |
| GTGTCCGCTTGGCATCGATA | MPK6-2 | 55% | 0 | 0 | 5 | * |
| GCGGGTGCGGGTCAATCAAA | MPK6-A | 60% | 0 | 6 | 0 | SL1 |
| GGAGGTCCTGGTCTAAACCA | MSH1 | 55% | 0 | 4 | 4 | SL1 |
| GGCGCGCGTCGAGGCGTTGG | MYB1 | 80% | 0 | 0 | 5 | * |
| GTAGCCCTGCTGTTGAAGAA | MYB5 | 50% | 0 | 5 | 2 | SL1 |
| GCTTTTCCCTGATGAAATTT | NbPDS | 35% | 0 | 6 | 0 | SL1 |
| GCCGTTAATTTGAGAGTCCA | NbPDS3 | 45% | 0 | 4 | 0 | SL1 |
| GTAGATGCAGAAGCTAGAGT | NtPDR6-A | 45% | 0 | 5 | 0 | SL1 |
| GTGGAATCATCAAACCAGGA | NtPDR6-B | 45% | 0 | 5 | 0 | SL1 |
| GCCGTTAATTTGAGAGTCCA | NtPDS-A | 45% | 0 | 4 | 0 | SL1 |
| GCTGCATGGAAAGATGATGA | NtPDS-B | 45% | 0 | 5 | 0 | SL1 |
| GAGATTGTTATTGCTGGTGC | NtPDS-C | 45% | 0 | 6 | 0 | SL1 |
| TCACCTTGCAAACATGGCGC | Os01g0891000 | 55% | 0 | 0 | 3 | * |
| GGCTATTGGATCGTGCACCA | Os02g0459600 | 55% | 0 | 5 | 0 | SL1 |
| GCTGAAAGATTTGACGTCCC | Os02g23823 | 50% | 0 | 3 | 0 | SL1 |
| TGCACCTTGTATTTGCAGTT | Os03g0126800-A | 40% | 0 | 1 | 3 | SL1 |
| AAATCCCAACTGCAAATACA | Os03g0126800-B | 35% | 0 | 0 | 0 | * |
| GGAGGCTCCATCGAATCGAT | Os03g0216800 | 55% | 0 | 4 | 4 | SL1 |
| CGCAAGACAGGGCACACCGG | Os03g0247300 | 70% | 0 | 4 | 0 | SL1 |
| CCTCGATCCTCCTCTGCAGA | Os04g0595000 | 60% | 0 | 3 | 0 | SL1 |
| GCGGAAGCCTCGACAGTCAC | Os04g0668400 | 65% | 0 | 5 | 2 | SL1 |
| AAGATCCATGAGTTCAAGAA | Os05g0312500 | 35% | 0 | 0 | 0 | * |
| AACCTGATCACCAACCCACT | Os05g0543000 | 50% | 0 | 4 | 0 | SL1 |
| AGAGGATCGGCTATAATACC | Os05g0591600 | 45% | 0 | 4 | 0 | SL1 |
| TTCATGCTCAGGCATCGCTA | Os06g0142000 | 50% | 0 | 3 | 4 | SL1 |
| CATGGGGAACTTGGACTTGG | Os06g0142100 | 55% | 0 | 5 | 2 | SL1 |
| AACGTGTTCGACCAGGAGGT | Os06g0275000-A | 55% | 0 | 3 | 0 | SL1 |
| GGGTATAGTACCAGACAGCA | Os06g0275000-B | 50% | 0 | 3 | 4 | SL1 |
| ATTTATCCGTTCATGTCGAT | Os07g0261200-A | 35% | 0 | 3 | 0 | SL1 |
| GATACTCACGATGGTGGCGC | Os07g0261200-B | 60% | 0 | 5 | 0 | SL1 |
| GCGAGCGAGAGTCATGGAGG | Os07g0409500-A | 65% | 0 | 4 | 0 | SL1 |
| GCACGCCAAGGTCGACGACA | Os07g0409500-B | 65% | 0 | 3 | 3 | SL1 |
| GTCGTCCATGGCCCCCAACC | Os07g0411300 | 70% | 0 | 5 | 0 | SL1 |
| GCGAGATCGAGCGATGGCGA | Os07g0625500-A | 65% | 0 | 4 | 0 | SL1 |
| GGCGCTCGCCAAGTTCGACA | Os07g0625500-B | 65% | 0 | 3 | 2 | SL1 |
| CCTCGCCGCCGTACGTGTAG | Os10g0413900-A | 70% | 0 | 2 | 0 | SL1 |
| GGGCGAGGGGATGGCGGCAC | Os10g0413900-B | 80% | 0 | 5 | 0 | SL1 |
| ACCTTGGATTCGGCGATGCC | Os10g0484800 | 60% | 0 | 3 | 0 | SL1 |
| GTGGTGGCAGCGCTGGTCGG | Os10g0548600 | 75% | 0 | 7 | 0 | SL1 |
| ATCTGGTGGGGCTGATCGGG | Os11g0549665 | 65% | 0 | 6 | 0 | SL1 |
| ATTGTGAACTATGCCAAGTC | Os12g0242700 | 40% | 0 | 4 | 0 | SL1 |
| GGAGGAGGACGCGGCCGCCG | OsAnP | 85% | 0 | 4 | 0 | SL1 |
| GTCGGCCGGCAGCCGGATGA | OsFTL1 | 75% | 0 | 0 | 5 | * |
| GGCTTGCTTACAACTGCAGA | OsFTL10 | 50% | 0 | 4 | 3 | SL1 |
| TGGCAAGGAGTTCCGTTCCT | OsFTL12 | 55% | 0 | 4 | 5 | SL1 |
| CGGTGCTGATGAAGGGATCC | OsFTL13 | 60% | 0 | 6 | 0 | SL1 |
| GTCACGAGGTAGGGATCCTT | OsFTL4 | 55% | 0 | 5 | 0 | SL1 |
| GACGCGCGGCTTGCCGGCGA | OsFTL5 | 80% | 0 | 4 | 0 | SL1 |
| ATCTTCACTAGCCATGTCAA | OsFTL6 | 40% | 0 | 7 | 0 | SL1 |
| TCGGCGCGTCGGCGCTGCTG | OsFTL7 | 80% | 0 | 3 | 5 | SL1 |
| CGGCGAACAGCCTGGTGCTG | OsFTL8 | 70% | 0 | 3 | 3 | SL1 |
| TATTGGTGGCACCGACCTGA | OsFTL9 | 55% | 0 | 5 | 3 | SL1 |
| GGCGGCCGCTCTTCCGCGGG | OsGSTU | 85% | 0 | 3 | 4 | SL1 |
| TGCTAAAACTGAAACTAGTA | OsMRP15 | 30% | 0 | 6 | 0 | SL1 |
| GGAAGGATGAAGATGGAGAT | PDS-A | 45% | 0 | 5 | 0 | SL1 |
| GTTGGTCTTTGCTCCTGCAG | PDS-B | 55% | 0 | 7 | 4 | SL1 |
| CACCTACATATAGGAGTGGG | PMS3 | 50% | 0 | 3 | 0 | SL1 |
| GCGGAGAACGACAGCCGGTC | ROC5 | 70% | 0 | 4 | 0 | SL1 |
| TGAAGCACCCCGCCGACATC | SlDNMT3B | 65% | 0 | 0 | 2 | * |
| TCTCCATATCTTCTTCCACA | SPL4 | 40% | 0 | 0 | 0 | * |
| GGCGATTGGGAGCCTCGGCG | SPP | 75% | 0 | 5 | 0 | SL1 |
| GCCTGTCCCTGCAGCATCCC | SWEET13 | 70% | 0 | 0 | 5 | * |
| GCTGAAGATGATGGGTCTCC | SWEET1A | 55% | 0 | 9 | 0 | SL1 |
| AGACGTACGAGTTTGTGCAG | TaINOX | 50% | 0 | 5 | 0 | SL1 |
| GGAGATTGGGTCCTGCGTGA | TaMLO | 60% | 0 | 3 | 0 | SL1 |
| TGTGTGCTTACAGCCATGGC | Waxy-A | 55% | 0 | 0 | 6 | * |
| GAGCCTCGAGTGCTGCCTGC | Waxy-B | 70% | 0 | 4 | 0 | SL1 |
| GTCTGAATCTTTTTCACTGC | Waxy-C | 40% | 0 | 0 | 4 | * |
| GCGCGCCACCTCGGCCGAAG | YSA | 80% | 0 | 0 | 5 | * |
| GGGAGAAGGAGACGGATCCC | ZmIPK1A | 65% | 0 | 7 | 0 | SL1 |
| TBP (Total base pairs); CBP (Consecutive base pairs); IBP (Internal base pairs); SL (stem looop); DSL (Despaired stem loop); * indicates that no stem loop is depaired; a these guide sequence were retreieved from CrisprGE database (Kaur,K., Tandon,H., Gupta, A.K. et al. 2015. CrisprGE: a central hub of CRISPR/Cas-based genome editing.Database Vol. 2015: article ID bav055; doi:10.1093/database/bav055). | | | | | | |

**Supplemental Table 2**. Public primers used for amplification of sgRNA cassettes.

| Primer ID | Primer sequence |
| --- | --- |
| Pro-F-SpeI | AAactagtTGGAATCGGCAGCAAAGG |
| sgRNA-R-BamHI | AAggatccATCCACTCCAAGCTCTTG |
| Pro-F-BamHI | AAggatccTGGAATCGGCAGCAAAGG |
| sgRNA-R-SmaI | AAcccgggATCCACTCCAAGCTCTTG |
| Pro-F-SmaI | AAcccgggTGGAATCGGCAGCAAAGG |
| sgRNA-R-PstI | AActgcagATCCACTCCAAGCTCTTG |
| Pro-F-PstI | AActgcagTGGAATCGGCAGCAAAGG |
| gRNA-R-EcoRI | AAgaattcATCCACTCCAAGCTCTTG |
| Pro-F-EcoRI | AAgaattcTGGAATCGGCAGCAAAGG |
| gRNA-R-EcoRV | AAgatatcATCCACTCCAAGCTCTTG |
| Pro-F-EcoRV | AAgatatcTGGAATCGGCAGCAAAGG |
| gRNA-R-HindIII | AAaagcttATCCACTCCAAGCTCTTG |
| Pro-F-HindIII | AAaagcttTGGAATCGGCAGCAAAGG |
| sgRNA-R-ClaI | AAatcgatATCCACTCCAAGCTCTTG |
| Pro-F-ClaI | AAatcgatTGGAATCGGCAGCAAAGG |
| sgRNA-R-SalI | AAgtcgacATCCACTCCAAGCTCTTG |
| Pro-F-SalI | AAgtcgacTGGAATCGGCAGCAAAGG |
| sgRNA-R-XhoI | AActcgagATCCACTCCAAGCTCTTG |
| Pro-F-XhoI | AActcgagTGGAATCGGCAGCAAAGG |
| sgRNA-R-AscI | AAggcgcgccATCCACTCCAAGCTCTTG |

**Supplemental Table 3**. Guide sequences and secondary structure characteristics of their sgRNAs

| Guide ID | Guide sequence | Target sites | G/C(%) | TBP | CBP | IBP | DSL |
| --- | --- | --- | --- | --- | --- | --- | --- |
| guide1 | CTTGGAACTGGGTGCTGTCC | LOC_Os07g35870 | 60% | 9 | 3 | 0 | SL1 |
| guide2 | GGCTCTGTGGACTGTGAACA | LOC_Os07g35870 | 55% | 7 | 7 | 0 | SL1 |
| guide3 | TAAGGTCAGGGTCTTGTGGT | LOC_Os08g04390 | 50% | 11 | 3 | 0 | SL1 |
| guide4 | CCTAAGTGATGCCACTCGCA | LOC_Os08g04390 | 55% | 11 | 6 | 0 | SL1 |
| guide5 | ATAGTTATGGAATATGCTGC | LOC_Os03g27280 | 35% | 10 | 5 | 0 | SL1 |
| guide6 | GTCGTTGAGGGGATATGGAG | LOC_Os03g27280 | 55% | 9 | 3 | 0 | SL1 |
| guide7 | AGATTGATGAAAATGTGCAG | LOC_Os10g41490 | 35% | 10 | 6 | 0 | SL1 |
| guide8 | GGTTTCGAGGGGGCCAATGG | LOC_Os10g41490 | 65% | 11 | 4 | 0 | SL1 |
| guide9 | GGCGAGACAGGCTCAATGAA | LOC_Os05g38140 | 55% | 11 | 4 | 0 | SL1 |
| guide10 | TCCAGAATCGAGCGCACCGC | LOC_Os05g38140 | 65% | 8 | 2 | 0 | SL1 |
| guide11 | ATCGTCATGGAGTACGCCGC | LOC_Os02g34600 | 60% | 12 | 5 | 0 | SL1 |
| guide12 | GGAGAAGTACGAGCTGCTCA | LOC_Os02g34600 | 55% | 11 | 3 | 0 | SL1 |
| guide13 | AGATTGACGAGAACGTGCAG | LOC_Os12g39630 | 50% | 12 | 4 | 0 | SL1 |
| guide14 | GCCGGCGGAAGCGATGGAGA | LOC_Os12g39630 | 70% | 9 | 6 | 0 | SL1 |
| guide15 | ATTGTCATGGAATATGCCTC | LOC_Os03g55600 | 40% | 11 | 5 | 0 | SL1 |
| guide16 | GAAGTTGGAGAATACACTGC | LOC_Os03g55600 | 45% | 10 | 4 | 0 | SL1 |
| guide17 | GCCATGAAGTACATCCCTCG | LOC_Os04g35240 | 55% | 3 | 3 | 0 | SL1 |
| guide18 | GAGCGGCGGGAGGCATGGAG | LOC_Os04g35240 | 75% | 11 | 6 | 0 | SL1 |
| guide19 | CGAAGACGATCGTGTCGGAG | LOC_Os04g31290 | 60% | 9 | 4 | 0 | SL1 |
| guide20 | GTCGATCATCGCCGACGCGG | LOC_Os04g31290 | 70% | 4 | 0 | 4 | * |
| guide21 | CGTCTGTTGGCAGAACTCAG | LOC_Os02g02480 | 55% | 4 | 0 | 4 | * |
| guide22 | CTTAATTTCTGTTAGGTTCT | LOC_Os07g35870 | 30% | 7 | 0 | 7 | * |
| guide23 | AGTACAAGTTTATTTTTTTC | LOC_Os07g35870 | 20% | 0 | 0 | 0 | * |
| guide24^a^ | GAAGTTGGTGACGGGACTTT | LOC_Os02g47220 | 50% | 18 | 14 | 0 | SL2 |
| TBP (Total base pairs); CBP (Consecutive base pairs); IBP (Internal base pairs); SL (stem looop); DSL (Despaired stem loop); * indicates that no stem loop is depaired. ^a^Ma, X. et al. A robustCRISPR/Cas9 system for convenient high-efficiency multiplex genome editing in monocot and dicot plants. Mol Plant doi: 10.1016/j.molp.2015.04.007 (2015). | | | | | | | |
